# Association of circulating proprotein convertase subtilisin/kexin type 9 concentration, prothrombin time and cardiovascular outcomes: a prospective cohort study

**DOI:** 10.1186/s12959-021-00344-0

**Published:** 2021-11-22

**Authors:** Jia Peng, Ming-Ming Liu, Hui-Hui Liu, Yuan-Lin Guo, Na-Qiong Wu, Qian Dong, Jie Qian, Ke-Fei Dou, Cheng-Gang Zhu, Jian-Jun Li

**Affiliations:** Cardiometabolic Medicine Center, State Key Laboratory of Cardiovascular Disease, Fu Wai Hospital, National Center for Cardiovascular Diseases, Chinese Academy of Medical Sciences and Peking Union Medical College, No 167 BeiLiShi Road, XiCheng District, Beijing, 100037 China

**Keywords:** PCSK9, Coagulation, PT, Atherosclerosis, Cardiovascular risks

## Abstract

**Background:**

Proprotein convertase subtilisin/kexin type 9 (PCSK9) is considered to have multiple roles in the development of atherosclerosis, which is recently reported to participate in the thrombotic process. We aimed to examine the relationship between PCSK9 concentration, coagulation indexes and cardiovascular events.

**Methods:**

A total of 2293 consecutive patients with angina-like chest pain and without lipid-lowering drugs treatment were enrolled and followed up for major adverse cardiovascular events (MACEs). Circulating PCSK9 concentration was determined by ELISA. The routine coagulation tests including activated partial thromboplastin time (APTT), prothrombin time (PT) and thrombin time were performed. The associations between PCSK9 concentration, routine coagulation indicators and MACEs were analyzed.

**Results:**

Patients with high PCSK9 levels had lower PT and APTT levels (all *p* <  0.05). However, PCSK9 concentration was only independently and negatively correlated with PT (β = − 0.115, *p* <  0.001). During a mean of 38.3 months, 186 (8.1%) MACEs were occurred. Multiple Cox regression analysis indicated high PCSK9 or low PT levels as risk factors related to MACEs. When the prognosis was analyzed by the combination of PCSK9 and PT levels, patients with high PCSK9 and low PT had higher incidence of MACEs compared to those with low PCSK9 and high PT.

**Conclusions:**

Our study firstly suggested that PCSK9 concentration was negatively correlated with plasma levels of PT. Furthermore, high PCSK9 and low PT were associated with MACEs and the combination of PCSK9 with PT had an addictive effect on predicting cardiovascular outcomes in patients with chest pain, which was useful for further subdivision of cardiovascular risks.

**Supplementary Information:**

The online version contains supplementary material available at 10.1186/s12959-021-00344-0.

## Introduction

Atherosclerotic cardiovascular disease (ASCVD) has become the one of major cause of mortality through the world. Currently, there is more convincing evidence that clotting activation resulting in hypercoagulability participates the formation of thrombi after rupture of atherosclerotic lesion, therefore, promoting development of coronary artery disease (CAD) [1–3]. Activated partial thromboplastin time (APTT), prothrombin time (PT) and thrombin time (TT) are contained in routine coagulation screening tests, reflecting activation of coagulation system and thrombotic state by quantified numbers, which is related to the formation of thrombosis and acute ischemic events [4]. APTT and PT are involved in intrinsic and extrinsic coagulation pathway respectively, which is associated not only with venous thromboembolism but also with arterial thrombosis. Moreover, a large-scale genome-wide association study (GWAS) has suggested that APTT and PT are relevant to the elevated risks of CAD in gene level [5]. However, the role of coagulation in predicting cardiovascular risks need to further evaluate.

Recently, proprotein convertase subtilisin/kexin type 9 (PCSK9) is considered as a major risk factor for CAD, which plays an important role in cholesterol metabolism by increasing the degradation of hepatic low density lipoprotein cholesterol receptors (LDLR), thereby resulting in increasing circulating low density lipoprotein cholesterol (LDL-C) levels [6, 7]. Thereafter, PCSK9 inhibitors have been recommended to apply in patients with severe hypercholesterolemia or with statin intolerance to reduce lipid-related cardiovascular risk according to guideline for the management of dyslipidemia [8]. More importantly, PCSK9 has been prompted to be associated with pro-coagulation to aggravate atherosclerosis, apart from regulating plasma LDL-C levels [9]. Furthermore, our previous study, found that circulating PCSK9 concentration was independently correlated with fibrinogen, a major coagulation-related protein in circulation, in stable CAD patients [10]. Nevertheless, there are no studies concerning the correlation between circulating PCSK9 concentration and routine coagulation tests and their relationship with major adverse cardiovascular events (MACEs) until now .

The aim of this present study was to explore the potential association between PCSK9 and routine coagulation indexes including APTT, PT and TT, and to evaluate the predictive ability of PCSK9 and routine coagulation indexes in MACEs among untreated patients with chest pain.

## Patients and methods

### Study design and population

The present study protocol was complied with Declaration of Helsinki and was approved by the hospital ethics review board (Fu Wai Hospital & National Center for Cardiovascular Diseases, Beijing, China). Every patient signed the informed written consent before enrolled in this study.

The flowchart of the current study was presented in supplementary Fig. S1. We consecutively recruited 2583 patients with angina-like chest pain from October 2012 to April 2018 and 2433 patients who completed the determination of PCSK9 and routine coagulation tests including APTT, PT and TT.

Because lipid-lowering medications could increase circulating PCSK9 concentration and anti-coagulation drugs could prolong clotting time, confounding study results, patients taking any lipid-lowering agents within 3 months and taking any anti-coagulation agents within 1 month prior to entering the study were excluded from the present study. In addition, patients with acute or heart failure (left ventricular ejection fraction, left ventricular ejection fraction < 45%), acute coronary syndrome, significant hematologic disorders (white blood cell count ≤3.0 × 10^9^/L or ≥ 20 × 10^9^/L), infectious or systematic inflammatory disease, thyroid dysfunction, severe liver and/or renal insufficiency and malignant disease were excluded from this study. 2348 patients were followed-up for MACEs. During a follow-up with a mean of 38.3 months, 91 patients lost to follow-up. Finally, a total of 2293 patients with angina-like chest pain were included in this study. All patients were divided according to the median of PCSK9 concentration (low/high PCSK9 groups) and further stratified by the mean of PT levels (low/high PT groups).

In the current study, 1447 patients were proven stable CAD according to following diagnostic criteria. Stable CAD was defined as typical angina-like chest pain, a positive treadmill exercise test (> 1 mm ST-segment depression), and stable obstructive lesion > 50% in at least 1 of the 3 major coronary arteries or major branches assessed by at least two independent senior interventional cardiologists who had no knowledge of the patients’ clinical characteristics and biochemical data. 1418 patients were diagnosed with hypertension. The definition of hypertension was repeated systolic and/or diastolic blood pressure ≥ 140 and/or ≥ 90 mmHg on 2 different occasions or if patients were currently taking antihypertensive drugs. 538 patients with diabetes was diagnosed as fasting serum glucose levels ≥6.99 mmol/L in multiple determinations or patients were being treated with oral hypoglycemic agents or insulin.

### Blood sample measurement

Fasting blood samples were obtained for all patients at admission and collected into EDTA-containing tubes. Plasma samples were prepared by centrifugation at 3500×g twice for 10 min each at 15–18 °C and were stored at − 80 °C until analysis. Plasma PCSK9 concentration was measured using a high-sensitivity, quantitative sandwich enzyme immunoassay (Quantikine ELISA, R&D Systems Europe Ltd) according to our previous study [11]. The lower limit of detection was 0.096 ng/mL. APTT testing was performed by the aPTT-SP® reagent with synthetic phospholipids and silica as activators (Instrumentation Laboratory, Bedford, USA) and PT testing was performed by the PT-Fibrinogen HS Plus® reagent (HemosIL® Instrumentation Laboratory. Bedford, USA) [12]. TT was performed by thrombin reagent. Plasma D dimer level was measured by Stago evolution (France). The plasma samples used to determine coagulation function test were not diluted and the results were automatically measured by the instruments of the Fu Wai hospital laboratory. The plasma levels of fibrinogen were quantitatively measured by the method of Clauss and a Stago autoanalyzer with STA Fibrinogen kit (Diagnostic Stago, Taverny, France). The concentrations of the plasma triglyceride (TG), total cholesterol (TC), high density lipoprotein cholesterol (HDL-C), LDL-C, apolipoprotein (apo) B, apoA-I and glucose were measured by automatic biochemistry analyser (Hitachi 7150, Japan). Hemoglobin A1C (HbA1C) was measured using Tosoh Automated Glycohemoglobin Analyser (HLC-723G8, Tokyo, Japan). The concentrations of high-sensitivity C-reactive protein (hsCRP) were determined using immunoturbidimetry (Beckmann Assay 360, Bera, CA, USA).

### Follow-up for MACEs

Every patient was followed-up by professional technicians through telephones every six month. They were blinded to the clinical data of all patients and the purpose of this study until death occurred or the last day of follow-up period. The present study was followed to the end of June 30, 2019. The following MACEs were defined as nonfatal myocardial infarction (MI), coronary revascularization, hospitalization for unstable angina, ischemic stroke and cardiovascular death. Nonfatal MI was diagnosed as typical angina-like chest pain with increased cardiac troponins or typical electrocardiogram changes of MI. Coronary revascularization included percutaneous coronary intervention and coronary artery bypass grafting later than 90 days after their discharge. Unstable angina was confirmed if patients appeared new-onset severe angina or rest angina with normal serum levels of cardiac enzymes that required admission. Ischemic stroke was described by acute cerebral infarction symptoms and cerebral diagnostic imaging.

### Statistical analysis

Clinical and laboratory data are expressed as mean ± standard deviation (normally distributed continuous data) or median with interquartile range (skewed distributed continuous data). Categorical variables were expressed as frequencies and percentages. Comparisons between categorical data were performed with Chi Squared tests, while continuous variables were assessed by unpaired t test (for normal distribution) or nonparametric Mann-Whitney test (for skewed distribution). To evaluate the association between log-transformed PCSK9 and other parameters, Pearson correlation analysis or spearman correlation analysis was used. Univariate linear regression analysis was used to analyze the relationship between APTT, PT and TT and lipid parameters including TC, TG, LDL-C and HDL-C. Stepwise multivariable linear regression analysis was performed to determine the relationship between PCSK9 and PT. To examine the relationship of PCSK9, coagulation-related indexes with MACEs, Kaplan-Meier curve and Cox regression analyses were used to calculate the event-free survival rates and hazard ratios (HR) for MACEs with 95% confidence interval (CI) of different PCSK9 and/ or PT subgroups. Statistical analysis was performed with Statistical Package for Social Sciences version 25.0. A *p* <  0.05 was considered to be statistically different.

## Results

### Baseline characteristics

In the present study, we consecutively enrolled 2293 patients (1387 males, mean age 55.6 ± 11.0 years) and the baseline characteristics including clinical data and laboratory parameters classified by the occurrence of MACEs were showed in Table 1. The circulating PCSK9 concentration ranged from 70.01 ng/mL to 551.97 ng/mL (median = 228.48 ng/mL). Additionally, the mean of APTT, PT and TT value were 36.25 ± 3.9 s, 12.75 ± 0.64 s and 16.40 ± 1.25 s. Patients with MACEs were older, had higher percentage of CAD and DM, lower percentage of family history of CAD, higher TC, LDL-C and HbA1C levels than that without MACEs (all *p* <  0.05). Meanwhile, a higher plasma of PCSK9 and fibrinogen concentration and a lower PT level were found in participants with MACEs compared to those without MACEs (all *p* <  0.05). There were no significant different regarding the proportion of male, the percentage of hypertension, smoking status, drinking status, the baseline levels of BMI, systolic and diastolic blood pressure, TG, HDL-C, glucose, hsCRP, APTT, TT, D dimer and platelet counts, and the usage of medications (all *p* > 0.05). Because hypercoagulability expresses itself in the short interval between the normal clotting time and the minimal clotting time and shortening of the PT is very sensitive to hypocoagulation but relatively insensitive to hypercoagulation, we defined the transformation of prothrombin time (PT-t) as the inverse of the difference between PT in patients and the minimal PT of normal plasma for further analysis. Interestingly, we found that PT-t levels were higher in patients with events than those without events (*p* <  0.05).

**Table 1:** The baseline characteristics in this study

	Overall	MACEs	non-MACEs	
	***n*** = 2293	***n*** = 186	***n*** = 2107	***p***
**Clinical data**				
Age (years)	55.62 ± 11.01	57.73 ± 9.8	55.44 ± 11.09	0.006
Male sex n(%)	1387 (60.5)	111 (59.7)	1276 (60.6)	0.813
BMI (kg/m2)	25.63 ± 3.38	25.95 ± 3.37	25.6 ± 3.38	0.175
CAD n(%)	1447 (63.1)	142 (76.3)	1305 (61.9)	< 0.001
DM n(%)	538 (23.5)	60 (32.3)	478 (22.7)	0.003
Hypertension n(%)	1418 (61.8)	125 (67.2)	1293 (61.4)	0.116
Smoking n(%)	788 (34.4)	63 (33.9)	725 (34.4)	0.882
Drinking n(%)	468 (20.4)	44 (23.7)	424 (20.1)	0.252
Family history of CAD n(%)	514 (22.4)	30 (16.1)	484 (23)	0.032
SBP (mmHg)	128.05 ± 18.01	129.57 ± 16.49	127.92 ± 18.13	0.231
DBP (mmHg)	79.59 ± 11.19	80.25 ± 11.98	79.53 ± 11.12	0.398
**Laboratory parameters**				
TC (mmol/L)	4.9 ± 1.09	5.09 ± 1.08	4.88 ± 1.09	0.014
TG (mmol/L)	(1.15,2.3)	1.65 (1.17,2.36)	1.58 (1.14,2.29)	0.598
HDL-C (mmol/L)	1.11 ± 0.35	1.11 ± 0.34	1.16 ± 0.37	0.074
LDL-C (mmol/L)	3.2 ± 1.0	3.35 ± 1.01	3.18 ± 1.0	0.030
Glu (mmol/L)	5.65 ± 1.88	5.84 ± 2.44	5.63 ± 1.83	0.145
HbA1C (%)	6.02 ± 1.0	6.24 ± 1.06	6.0 ± 1.0	0.001
hsCRP (mg/L)	1.37 (0.72,2.46)	1.65 (0.77,2.89)	1.36 (0.72,2.46)	0.192
PCSK9 (ng/mL)	228.48 (189,275.5)	246.69 (208.92,297.11)	227.24 (187.33,274.18)	< 0.001
**Coagulation indexes**				
APTT (s)	36.25 ± 3.9	36 ± 3.91	36.27 ± 3.89	0.374
PT (s)	12.75 ± 0.64	12.66 ± 0.58	12.76 ± 0.64	0.032
PT-t (1/s)	0.83 (0.63.1.25)	0.91 (0.70,1.25)	0.83 (0.63,1.25)	0.028
TT (s)	16.4 ± 1.25	16.28 ± 1.55	16.41 ± 1.22	0.170
Fib (g/L)	3.06 ± 0.73	3.19 ± 0.85	3.05 ± 0.71	0.013
D dimer (μg/mL)	0.31 (0.24,0.41)	0.31 (0.23,0.45)	0.31 (0.24,0.41)	0.531
Platelet(10^12)	216.79 ± 55.66	212.69 ± 49.81	217.15 ± 56.14	0.295
**Medication**				
Aspirin/Clopidogrel n(%)	748 (32.6)	72 (38.7)	676 (32.1)	0.065
ACEI/ARB n(%)	383 (16.7)	34 (18.3)	349 (16.6)	0.548

*BMI* body mass index, *CAD* coronary artery disease, *DM* diabetes mellitus, *SBP* systolic blood pressure, *DBP* diastolic blood pressure, *TC* total cholesterol, *TG* triglyceride; HDL-C, high-density lipoprotein cholesterol, *LDL-C* low-density lipoprotein cholesterol, *Glu* glucose, *HbA1C* hemoglobin A1C, *hs-CRP* high-sensitivity C-reactive protein, *PCSK9* proprotein convertase subtilisin/kexin type 9, *APTT* activated partial thromboplastin time, *PT* prothrombin time, *PT-t* transformation of prothrombin time (the inverse of the difference between PT and the minimal PT of normal plasma), *TT* thrombin time, *Fib* fibrinogen, *ACEI* angiotensin converting enzyme inhibitors, *ARB* angiotensin receptor blockers. *p* < 0.05 suggested significant difference

Next, we divided all patients into two groups according to PCSK9 median to analyze baseline characteristic data and coagulation parameters, as summarized in Table 2. The data showed that age, gender, smoking status, drinking status, diastolic blood pressure and family history of CAD were statistical difference between high and low PCSK9 subgroups (all *p* < 0.05). Furthermore, patients with high PCSK9 levels trended to have higher TC, TG, HDL-C, LDL-C, HbA1C and hsCRP levels (all *p* < 0.05). In addition, APTT and PT were lower and PT-t, fibrinogen, d dimer and platelet counts were higher in patients with high PCSK9 levels than those with low PCSK9 levels (all *p* < 0.05). However, no significant difference with regard to variables including BMI, the percentage of CAD, diabetes mellitus and hypertension, systolic and diastolic blood pressure, glucose, TT, and the usage of medications (all *p* > 0.05).

**Table 2:** The clinical and biochemical data according to PCSK9 levels

Variables		PCSK9 (ng/mL)		***p***
	Overall	< 228.48	> 228.48	
	***n*** = 2293	***n*** = 1146	***n*** = 1147	
**Clinical data**				
Age (years)	55.62 ± 11.01	54.92 ± 11.12	56.32 ± 10.86	0.002
Male sex n(%)	1387 (60.5)	795 (69.4)	592 (51.6)	< 0.001
BMI (kg/m^2^)	25.63 ± 3.38	25.76 ± 3.39	25.49 ± 3.37	0.054
CAD n(%)	1447 (63.1)	723 (63.1)	724 (63.1)	0.987
DM n(%)	538 (23.5)	257 (22.4)	281 (24.5)	0.242
Hypertension n(%)	1418 (61.8)	710 (62.0)	708 (61.7)	0.910
Smoking n(%)	788 (34.4)	422 (36.8)	366 (31.9)	0.013
Drinking n(%)	468 (20.4)	261 (22.8)	207	0.005
Family history of CAD n(%)	514 (22.4)	236 (20.6)	278 (24.2)	0.036
SBP (mmHg)	128.05 ± 18.01	128.58 ± 17.97	127.52 ± 18.04	0.161
DBP (mmHg)	79.59 ± 11.19	80.08 ± 11.21	79.1 ± 11.16	0.035
**Laboratory parameters**				
TC (mmol/L)	4.9 ± 1.09	4.68 ± 0.96	5.12 ± 1.17	< 0.001
TG (mmol/L)	(1.15,2.3)	1.53 (1.11,2.23)	1.64 (1.19,2.35)	0.012
HDL-C (mmol/L)	1.11 ± 0.35	1.09 ± 0.36	1.14 ± 0.33	0.001
LDL-C (mmol/L)	3.2 ± 1	2.99 ± 0.88	3.41 ± 1.08	< 0.001
Glu (mmol/L)	5.65 ± 1.88	5.6 ± 2.1	5.69 ± 1.64	0.264
HbA1C (%)	6.02 ± 1.0	5.94 ± 0.94	6.09 ± 1.06	< 0.001
hsCRP (mg/L)	1.37 (0.72,2.46)	1.32 (0.71,2.46)	1.47 (0.73,2.63)	0.009
**Coagulation indexes**				
APTT (s)	36.25 ± 3.9	36.46 ± 3.86	36.03 ± 3.92	0.008
PT (s)	12.75 ± 0.64	12.84 ± 0.63	12.67 ± 0.63	< 0.001
PT-t (1/s)	0.83 (0.63.1.25)	0.77 (0.59,1.11)	0.83 (0.67,1.43)	< 0.001
TT (s)	16.4 ± 1.25	16.38 ± 1.08	16.42 ± 1.39	0.386
Fib (g/L)	3.06 ± 0.73	2.98 ± 0.64	3.15 ± 0.79	< 0.001
D dimer (μg/mL)	0.31 (0.24,0.41)	0.3 (0.24,0.39)	0.32 (0.24,0.43)	0.011
Platelet(10^12)	216.79 ± 55.66	211.32 ± 54.48	222.25 ± 56.31	< 0.001
**Medication**				
Aspirin/Clopidogrel n(%)	748 (32.6)	379 (33.1)	369 (32.2)	0.646
ACEI/ARB n(%)	383 (16.7)	193 (16.8)	190 (16.6)	0.859

*PCSK9* proprotein convertase subtilisin/kexin type 9, *BMI* body mass index, *CAD* coronary artery disease, *DM* diabetes mellitus, *SBP* systolic blood pressure, *DBP* diastolic blood pressure, *TC* total cholesterol, *TG* triglyceride, *HDL-C* high-density lipoprotein cholesterol, *LDL-C* low-density lipoprotein cholesterol, *Glu* glucose, *HbA1C* hemoglobin A1C, *hs-CRP* high-sensitivity C-reactive protein, *APTT* activated partial thromboplastin time, *PT* prothrombin time, *PT-t* transformation of prothrombin time (the inverse of the difference between PT and the minimal PT of normal plasma), *TT* thrombin time, *Fib* fibrinogen, *ACEI* angiotensin converting enzyme inhibitors, *ARB* angiotensin receptor blockers. *p* < 0.05 suggested significant difference

### Associations of PCSK9 and coagulation-related parameters

**Fig. 1:**
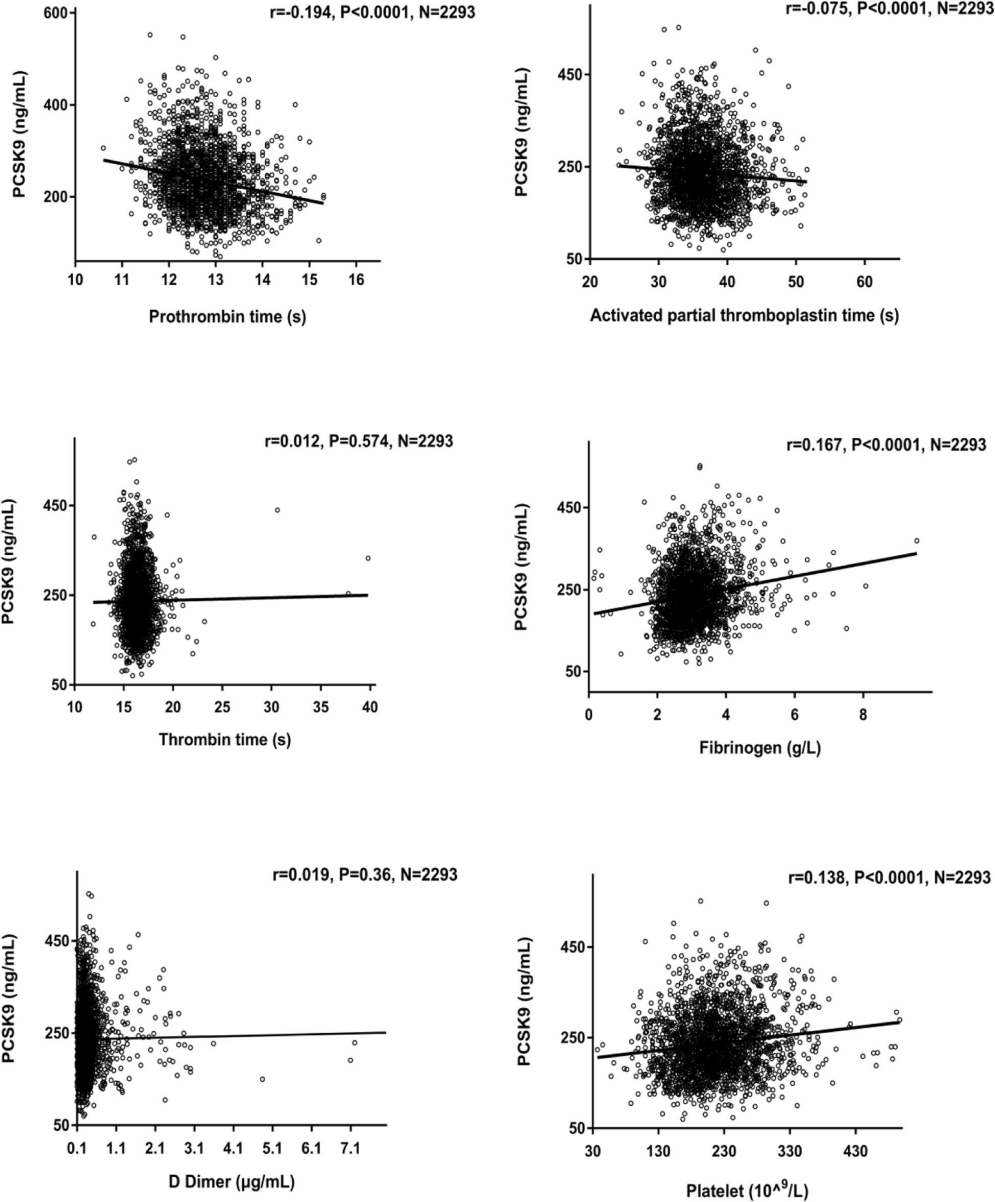
Association of PCSK9 and coagulation-related indicators in all patients (*N* = 2293). *p* < 0.05, PCSK9, proprotein convertase subtilisin/Kexin type 9

Pearson or spearman correlation analysis was performed in this study to clarify the association with log-transformed PCSK9 levels with parameters in all patients (Table S1). Consistent with previous results, our data showed that circulating PCSK9 concentration was associated with age, TC, HDL-C, LDL-C, glucose, HbA1C and hsCRP levels (r = 0.073, *p* < 0.001; r = 0.269, *p* < 0.001; r = 0.111, *p* < 0.001; r = 0.259, *p* < 0.001; r = 0.065, *p* = 0.002; r = 0.113, *p* < 0.001; r = 0.107, *p* < 0.001, respectively, Table S1). Moreover, we observed a negative correlation of APTT and PT, a positive correlation of fibrinogen and platelet counts with plasma levels of PCSK9 levels in the present study (r = − 0.075, *p* < 0.001; r = − 0.194, *p* < 0.001; r = 0.167, *p* < 0.001; r = 0.138, *p* < 0.001, respectively, Table S1 and Fig. 1). Additionally, a positive relation of PT-t with PCSK9 was found in all patients (r = 0.180, *p* < 0.001, Table S1). However, no significant correlation between PCSK9 levels and body mass index (BMI), systolic and diastolic blood pressure, TG, TT and D dimer (all *p* > 0.05).

In order to eliminate impact of potential association with circulating PCSK9 concentration and PT with age, gender, hypertension, diabetes mellitus, smoking status, BMI, a stepwise multivariate linear regression analysis was used to determine the relationship between PCSK9 levels and PT after adjusting for these confounders (Table 3). The results indicated that a negative association with PCSK9 levels and PT remained significant after adjustment for age, gender, hypertension, diabetes mellitus, CAD, smoking status, BMI (β = − 0.189, *p* < 0.001, Table 3). Even if adding TG, TC, HDL-C, LDL-C, glucose, HbA1C and hsCRP to multivariate linear regression model, the negative correlation of PCSK9 levels and PT remained statistically significant (β = − 0.135, *p* < 0.001). Due to APTT, TT, fibrinogen and D dimer reflect the coagulation status in vivo, and then we further added these parameters to adjust. Similarly, it showed that plasma PCSK9 level and PT still remained significant correlation in all patients (β = − 0.115, *p* < 0.001). Likewise, it was worth noting that PT-t levels were independently and positively associated with higher concentration of PCSK9 after adjusting for multiple confounders in stepwise multivariate linear regression analysis (β = 0.085, *p* < 0.001, Table 3). However, the significant difference between PCSK9 and APTT was disappeared in multivariable regression stepwise model (*p* = 0.367, Table S2).

**Table 3:** Independent determinants of PT or PT-t to log-transformed PCSK9

	Independent variable PT		Independent variable PT-t	
	Standardized regression coefficients β	***p***	Standardized regression coefficients β	***p***
Model 1^a^	−0.192	**< 0.001**	0.139	**< 0.001**
Model 2^b^	− 0.19	**< 0.001**	0.137	**< 0.001**
Model 3^c^	−0.189	**< 0.001**	0.136	**< 0.001**
Model 4^d^	−0.142	**< 0.001**	0.105	**< 0.001**
Model 5^e^	−0.135	**< 0.001**	0.100	**< 0.001**
Model 6^f^	−0.135	**< 0.001**	0.100	**< 0.001**
Model 7^g^	−0.115	**< 0.001**	0.085	**< 0.001**

The multivariable regression stepwise models are shown. The log-transformed PCSK9 is the dependent variable.

^a^Adjusted for age and sex

^b^Adjusted for age, sex, hypertension and diabetes, CAD

^c^Adjusted for age, sex, hypertension, diabetes, CAD, smoking status and BMI

^d^Adjusted for age, sex, hypertension, diabetes, CAD, smoking status, BMI, TG, TC, HDL-C and LDL-C

^e^Adjusted for age, sex, hypertension, diabetes, CAD, smoking status, BMI, TG, TC, HDL-C, LDL-C, glucose and HbA1C

^f^Adjusted for age, sex, hypertension, diabetes, CAD, smoking status, BMI, TG, TC, HDL-C, LDL-C, glucose, HbA1C and hsCRP

^g^Adjusted for age, sex, hypertension, diabetes, CAD, smoking status, BMI, TG, TC, HDL-C, LDL-C, glucose, HbA1C, hsCRP, fibrinogen, D dimer, platelet, APTT and TT. PT, prothrombin time; PT-t, transformation of prothrombin time (the inverse of the difference between PT and the minimal PT of normal plasma), *PCSK9* proprotein convertase subtilisin/Kexin type 9, *CAD* coronary artery disease, *BMI* body mass index, *TG* triglyceride, *TC* total cholesterol, *HDL-C* high-density lipoprotein cholesterol, L*DL-C* low-density lipoprotein cholesterol, *HbA1C* hemoglobin A1C, *hsCRP* high-sensitivity C-reactive protein, *ESR* erythrocyte sedimentation rate, *APTT* activated partial thromboplastin time, *TT* thrombin time

### Associations of PCSK9, PT and MACEs

**Fig. 2:**
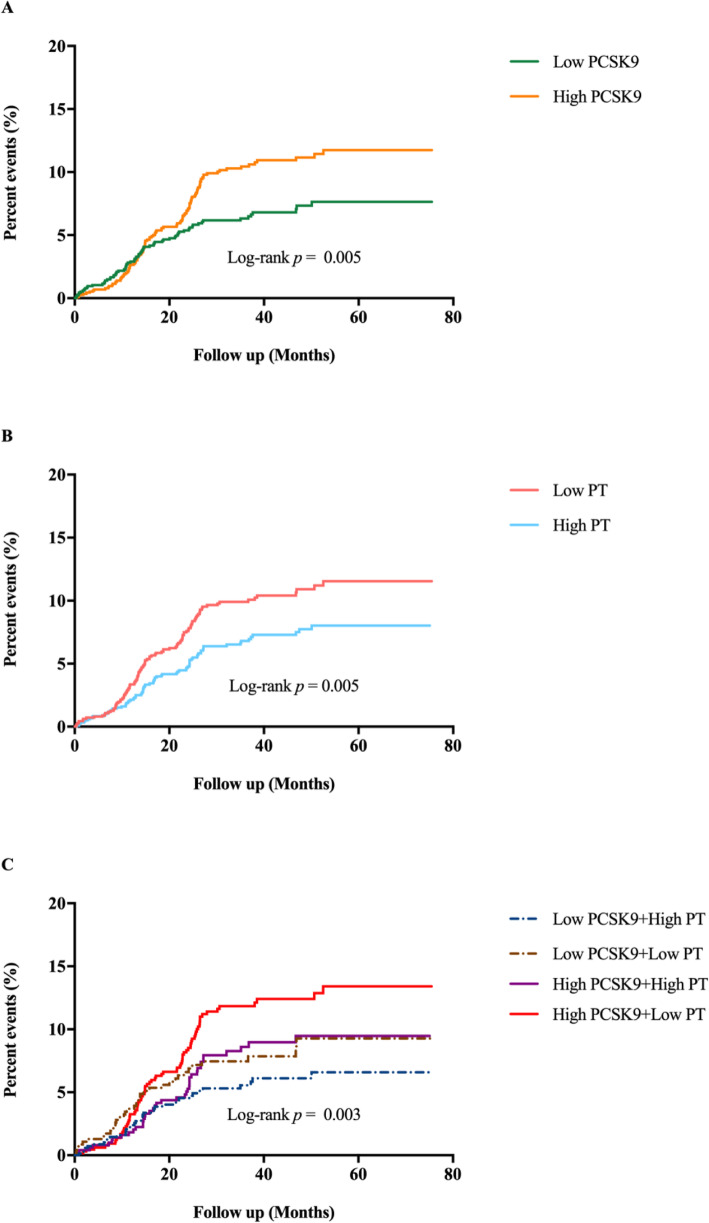
Kaplan-Meier analysis according to different PCSK9, PT levels and the combination of PCSK9 and PT status. PCSK9, proprotein convertase subtilisin/kexin type 9; PT, prothrombin time

**Fig. 3:**
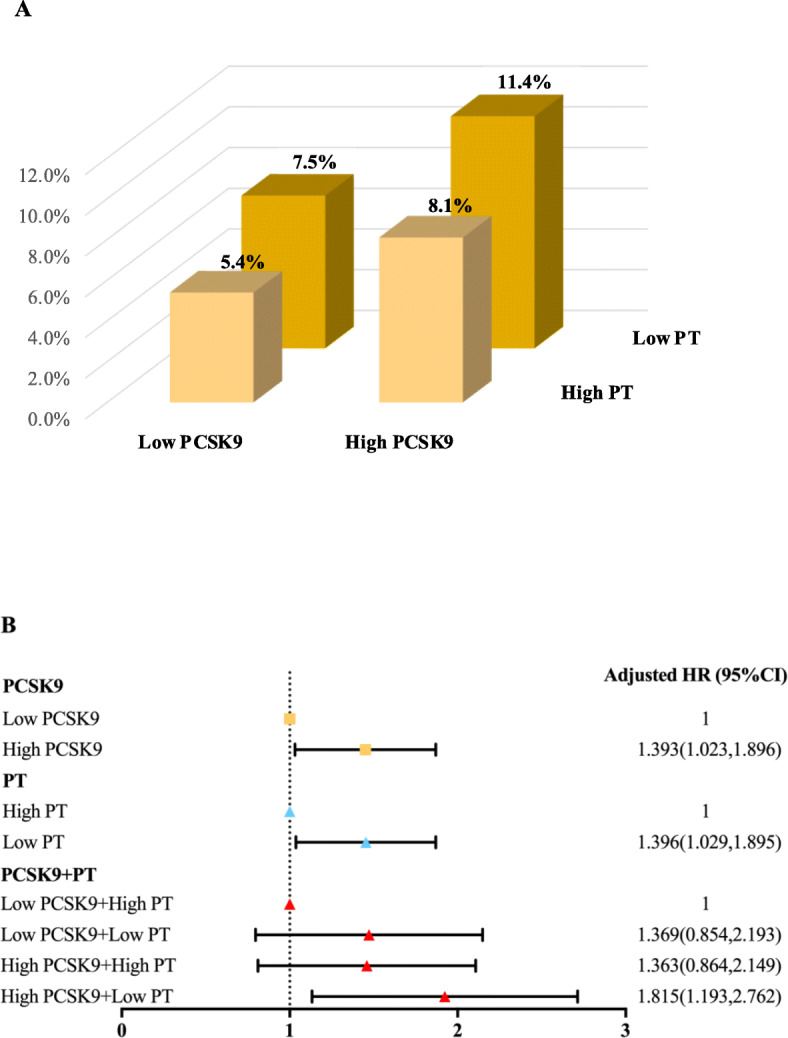
**A**. The percentage of MACEs in different subgroups according to the combination of PCSK9 and PT. **B**. Multiple Cox regression analysis in different subgroups. Adjusted model was adjusted for age, gender, CAD, diabetes, family history of CAD, TC, LDL, HbA1C, fibrinogen. MACEs, major adverse cardiovascular events; PCSK9, proprotein convertase subtilisin/kexin type 9; PT, prothrombin time; HR, hazard ratio; CI, confidence interval; CAD, coronary artery disease; TC, total cholesterol; HDL-C, LDL-C, low-density lipoprotein cholesterol; HbA1C, hemoglobin A1C

One hundred eighy-six MACEs including 9 died of cardiovascular diseases, 55 underwent coronary revascularization, 3 suffered nonfatal MI, 16 had ischemic stroke and 103 experienced hospitalizations because of unstable angina pectoris, were developed in a mean follow-up of 38.3 months in the current study. Kaplan Meier curve indicated that patients in high PCSK9 or low PT groups had higher incidence of MACEs compared with patients in low PCSK9 or high PT groups (all *p* < 0.05, Fig. 2A and 2B). When the event-free survival rate classified into 4 subgroups by the combination of PCSK9 and PT, high PCSK9 + low PT group was more likely to have significantly lower event-free survival rate compared to low PCSK9 + high PT group (the reference group, Fig. 2C, *p* < 0.05), whereas there was no significant difference in low PCSK9 + low PT and high PCSK9 + high PT groups compared with the reference group (all *p* > 0.05). Moreover, the incidence of MACEs was 5.4% (low PCSK9 + high PT), 7.5% (low PCSK9 + low PT), 8.1% (high PCSK9 + high PT group) and 11.4% (high PCSK9 + low PT group) in ascending order (Fig. 3A, *p* = 0.001). Multivariate Cox regression analysis showed that subjects in high PCSK9 or low PT groups had 1.393-fold (95%CI: 1.023–1.896) and 1.396-fold (95%CI: 1.029–1.895) higher risk of events than those in low PCSK9 or high PT groups (Table 4). Subsequently, we observed the combined impact of PCSK9 and PT on future cardiovascular events, and found that the multivariable-adjusted HR with 95%CI in high PCSK9 + low PT group was 1.815 (1.193–2.762) compared to the reference group. Similarly, we classified all patients into high or low PT-t groups based on the median of PT-t and found that patients in high PT-t group had higher risk of MACEs compared with those in low PT-t group (adjusted HR: 1.396, 95%CI: 1.029–1.895, *p* = 0.032, Table S3). When combined the PCSK9 levels and PT-t status, a higher incidence of cardiovascular events was occurred in high PCSK9 + high PT-t group compared with low PCSK9 + low PT-t group (adjusted HR: 1.815, 95%CI: 1.193–2.762, *p* = 0.005, Table S3).

**Table 4:** Cox regression analysis of PCSK9, PT status with MACEs

Variables	Events/subjects	HR(95%CI)			
		Crude model	***p***	Adjusted model	***p***
**PCSK9**					
Low PCSK9	72/1146	1 (Reference)	/	1 (Reference)	/
High PCSK9	114/1147	1.526 (1.136,2.049)	0.005	1.393 (1.023,1.896)	0.035
**PT**					
High PT	77/1178	1 (Reference)	/	1 (Reference)	/
Low PT	109/1115	1.512 (1.129,2.024)	0.006	1.396 (1.029,1.895)	0.032
**PCSK9 + PT**					
Low PCSK9 + High PT	37/682	1 (Reference)	/	1 (Reference)	/
Low PCSK9 + Low PT	35/464	1.415 (0.891,2.246)	0.141	1.369 (0.854,2.193)	0.192
High PCSK9 + High PT	40/496	1.43 (0.914,2.236)	0.117	1.363 (0.864,2.149)	0.183
High PCSK9 + Low PT	74/651	2.049 (1.381,3.041)	< 0.001	1.815 (1.193,2.762)	0.005

Adjusted model was adjusted for age, gender, CAD, diabetes, family history of CAD, TC, LDL, HbA1C, fibrinogen. MACEs, major adverse cardiovascular events; PCSK9, proprotein convertase subtilisin/kexin type 9; *PT* prothrombin time, *HR* hazard ratio, *CI* confidence interval, *CAD* coronary artery disease, *TC* total cholesterol, *HDL-C, LDL-C* low-density lipoprotein cholesterol, *HbA1C* hemoglobin A1C. *p* < 0.05 suggested significant difference

## Discussion

In the present study, we evaluated the association of circulating PCSK9 concentration with indexes of routine coagulation tests and cardiovascular events. The major findings of our study were that circulating PCSK9 concentration was independently and negatively correlated with PT, and the combination of high PCSK9 and low PT was associated with elevated cardiovascular outcomes, which might further provide novel information with regard to PCSK9 and hypercoagulability in cardiovascular risks.

As commonly recognized, PCSK9, a novel regulator of cholesterol metabolism, takes part in atherosclerosis, leading to the occurrence of CAD. Our previous studies had observed a higher circulating PCSK9 levels in patients with stable CAD patients and PCSK9 was significantly and positively associated with severity of coronary and adverse cardiovascular prognosis [13, 14]. Recently, FOURIER trial and ODYSSEY OUTCOMES trial have confirmed that PCSK9 inhibitors reduce major adverse cardiovascular and cerebrovascular events in patients with ASCVD compared with the placebo group [15, 16]. Interestingly, PCSK9 has been known to have various effects beyond LDL-C, which has been reported to be related to hypercoagulability and promote thrombosis to accelerate atherosclerosis [9]. Moreover, prior studies indicated that plasma PCSK9 levels were positively correlated with coagulation fibrinogen and platelet indices [10, 17]. Additionally, several animal studies using PCSK9 over expressing or PCSK9 knockout mice model found that an interesting phenomenon related to PCSK9 and hypercoagulable state, platelet activation and carotid artery thrombosis [18, 19]. However, there is no clinical data for explore the relationship between the PCSK9 and indicators of regular coagulation including APTT, PT and TT.

In the current study, we, for the first time, found that PCSK9 was only independently correlated with lower PT levels in patients with angina-like chest pain who took no lipid-lowering therapy. In order to further support the association of PCSK9 wIthink hypercoagulable state, we analyzed the relation of PCSK9 with PT-t and found a similar result. An increasing study have provided that hypercoagulability plays a crucial role in atherosclerosis contributing to both atherosclerotic plaque development and acute thrombotic complications, which is considered as a primary cause of morbidity and mortality of patients with CAD [20–22]. Furthermore, it is well known that coagulation factors can trigger non-hemostatic proatherogenic effect [23]. PT, the major component of routine coagulation tests, is a measure of the integrity of the extrinsic and final common pathways of the pro-coagulant cascade, which is recognized as a risk factor of CAD [24, 25]. A GWAS study for PT conducted in 2583 participants and identified genome-wide significant associations of the *F7* and *PROCR/EDEM2* regions with PT, of which may relate to the risk of CAD by assessment of these gene expression and CAD database comprised 22,233 cases of CAD and 64,762 controls of European ancestry [5]. Nevertheless, the role of PT in predicting cardiovascular prognosis needs to further investigate in different population. What’s more, whether there is an additive effect of two measures on MACEs remains to explore.

In this study, we evaluated the predictive ability of routine coagulation indexes including APTT, PT, and TT in MACEs. Firstly, we found that only lower PT levels (or higher PT-t levels), reflecting hypercoagulable state, were related to adverse cardiovascular outcomes in all patients. It is known that increasing or decreasing circulating concentration and activation or inhibition of clotting factors involved in extrinsic pathways of coagulation cascade will lead to a shortening or extension of PT. Activated of TF and factor VII (FVII) are the initial and pivotal step to trigger extrinsic coagulation pathway, which is different from intrinsic coagulation pathway reflected by APTT [26, 27]. When vascular injury exposes plasma to a variety of TF-expressing cells, zymogen FVII binds to TF and rapidly converts to FVIIa by limited proteolysis, thereby generating the active TF-FVIIa complex to greatly increase the enzymatic activity of FVIIa [28]. Moreover, it was showed that recombinant human FVIIa administration to individuals with liver disease resulted in a transient shortening of PT [29]. Therefore, circulating TF and FVII levels reduce or increase and TF and FVII activity is been inhibited or activated, the value of PT would be changed. Notably, tissue factor pathway inhibitor (TFPI), an important factor in the extrinsic pathway of coagulation cascade, could result in a negative feedback on the formation of TF/FVIIa complexes to shut down the extrinsic activation of coagulation in vivo. Furthermore, accumulating evidence of animal and clinical studies showed that TF and TFPI were closely associated with atherosclerosis and CAD [30–35]. Meanwhile, data on the prognostic value of TF activity or TF suggested that systemic TF activity had a harmful prognostic value in patients with AMI from a prospective study recruited 174 patients with unstable angina pectoris and 112 patients with acute myocardial infarction followed for a mean period of 3.26 years [35], and from a prognostic study with 1146 patients stable angina pectoris and 523 patients with acute coronary syndrome followed over a mean period of 2.3 years [34]. These results supported our finding of lower PT or higher PT-t associated with MACEs.

Nonetheless, a large-scaled clinical trial with 2 years follow-up and revealed that elevated PT was an independent risk predictor for all-cause mortality in 2734 acute MI patients (adjusted HR, 4.04; 95%CI, 2.83–5.7) [36], which seemed to be inconsistent with our results. It was worth to noting that the median of PT in acute MI patients was 13.85 seconds which was larger than our patients and meant that they had a greater risk of bleeding. In addition, the endpoint event observed of this study was all-cause of death but did not further analyze the association of PT with cardiovascular death. Therefore, our study firstly found that the hypercoagulable state presented by low PT level or high PT-t level were an independently risk factor of cardiovascular events.

As well-known, PCSK9 levels have been proposed to predict cardiovascular outcomes in different population [37]. Similarly, our data also found that circulating levels of PCSK9 were significantly associated with MACEs in patients with chest pain. Of note, our study further revealed that the combination of high PCSK9 and low PT had better predictive ability in cardiovascular outcomes in entire population after adjusting for multiple risk factors, which provided a new connection of lipid metabolism and coagulation in ASCVD. PCSK9 may have impact on extrinsic coagulation cascade via altering the levels and activity of TF and/or FVII. Meanwhile, it has been reported that dyslipidemia and hypercoagulable state play critical role in atherosclerosis and cardiovascular risks [35, 37]. Furthermore, the link between the two abnormal states has been described, especially in the relation of LDL abnormality and extrinsic coagulation dysfunction [30, 38]. Importantly, PCSK9 directly and remarkably has currently reported to reduce circulating LDL-C levels by mediating lysosomal degradation of hepatic LDLR and has impacts on thrombosis [8]. Hence, the combination of PCSK9 and PT (or PT-t) had an additive impact on the incidence of cardiovascular events in patients with angina-like chest pain, which was benefited for cardiovascular risk stratification.

There were some limitations in this study. First, the sample size recruited in a single center had effect on the generalizability of the results. Second, the cross-sectional design of this study could not exclude residual confoundings. Third, the current study only observed the association between PCSK9 and PT, but did not investigate the relationship of PCSK9 with several important extrinsic clotting factors such as aforementioned TF and FVII. Additionally, the possible causal association of PCSK9 and PT was not been confirmed because this was an observational and prospective study. Finally, we could not examine the changes of PCSK9 and PT, and these impacts on predicting MACEs during follow-up.

## Conclusions

The present study for the first time showed a negative relation of PCSK9 with PT and found that the combination of PCSK9 and PT was significantly associated with adverse cardiovascular outcomes, suggesting that PCSK9 may induce and/or promote hypercoagulability.The combination of these two measures may be useful to further identify cardiovascular risk stratification.

## Supplementary Information

**Additional file 1: Table S1.** Correlation analysis between log-transformed PCSK9 and related-parameters in all patients (N=2293). **Table S2.** Multiple linear analysis of PCSK9 and coagulation-related indexes. **Table S3.** Cox regression analysis of PCSK9, PT-t status with MACEs. **Figure S1.** The flowchart of this study. PCSK9, proprotein convertase subtilisin/kexin type 9; MACEs, major adverse cardiovascular events.

## Data Availability

The datasets used and analyzed during the current study are available from the corresponding author on reasonable request.

## Abbreviations

ASCVD: Atherosclerotic cardiovascular disease
CAD: Coronary artery disease
APTT: Activated partial thromboplastin time
PT: Prothrombin time
TT: Thrombin time
PCSK9: Proprotein convertase subtilisin/kexin type 9
LDLR: Low density lipoprotein cholesterol receptor
LDL-C: Low density lipoprotein cholesterol
MACEs: Major adverse cardiovascular events
TG: Triglyceride
TC: Total cholesterol
HDL-C: High density lipoprotein cholesterol
HbA1C: Hemoglobin A1C
hsCRP: High-sensitivity C-reactive protein
MI: Myocardial infarction
HR: Hazard ratios
CI: Confidence interval
PT-t: The transformation of prothrombin time
TF: Tissue factor
TFPI: Tissue factor pathway inhibitor
FVII: Factor VII
