# Supplementary material for: Association of circulating proprotein convertase subtilisin/kexin type 9 concentration, prothrombin time and cardiovascular outcomes: a prospective cohort study
Source: Thromb J. 2021 Nov 22;19:90. doi: 10.1186/s12959-021-00344-0 (PMC8607723; doi:10.1186/s12959-021-00344-0)
Supplement: Supplementary file 1 — Additional file 1: Table S1. Correlation analysis between log-transformed PCSK9 and related-parameters in all patients (N=2293). Table S2. Multiple linear analysis of PCSK9 and coagulation-related indexes. Table S3. Cox regression analysis of PCSK9, PT-t status with MACEs. Figure S1. The flowchart of this study. PCSK9, proprotein convertase subtilisin/kexin type 9; MACEs, major adverse cardiovascular events. [file 12959_2021_344_MOESM1_ESM.docx]

**Supplementary**

**Table S1. Correlation analysis between log**-**transformed PCSK9 and related**-**parameters in all patients (N=2293).**

|  | **All patients (N=2293)** | |
| --- | --- | --- |
| **Variables** | **r** | ***p*** |
| Age | 0.073 | <0.001 |
| BMI | -0.026 | 0.222 |
| SBP | -0.012 | 0.568 |
| DBP | -0.040 | 0.056 |
| TC | 0.269 | <0.001 |
| TG | 0.030 | 0.146 |
| HDL-C | 0.111 | <0.001 |
| LDL-C | 0.259 | <0.001 |
| Glucose | 0.065 | 0.002 |
| HbA1C | 0.113 | <0.001 |
| hsCRP | 0.107 | <0.001 |
| APTT | -0.075 | <0.001 |
| PT | -0.194 | <0.001 |
| PT-t | 0.180 | <0.001 |
| TT | 0.012 | 0.574 |
| Fib | 0.167 | <0.001 |
| D Dimer | 0.019 | 0.360 |
| Platelet | 0.138 | <0.001 |

PCSK9, proprotein convertase subtilisin/kexin type 9; BMI, body mass index; SBP, systolic blood pressure; DBP, diastolic blood pressure; TC, total cholesterol; TG, triglyceride; HDL-C, high-density lipoprotein cholesterol; LDL-C, low-density lipoprotein cholesterol; HbA1C, hemoglobin A1C; hs-CRP, high-sensitivity C-reactive protein; ESR, erythrocyte sedimentation rate; APTT, activated partial thromboplastin time; PT, prothrombin time; PT-t, transformation of prothrombin time (the inverse of the difference between PT and the minimal PT of normal plasma); TT, thrombin time; Fib, fibrinogen.

**Table S2. Multiple linear analysis of PCSK9 and coagulation-related indexes.**

| **Coagulation-related indexes** | **Standardized coefficients β** | ***p*** |
| --- | --- | --- |
| PT | -0.115 | < 0.001 |
| PT-t | 0.085 | < 0.001 |
| APTT | -0.019 | 0.367 |
| TT | 0.026 | 0.196 |
| Fib | 0.074 | 0.001 |
| platelet | 0.036 | 0.085 |

Adjusted for age, sex, hypertension, diabetes, CAD, smoking status, BMI, TG, TC, HDL-C, LDL-C, glucose, HbA1C, hsCRP, fibrinogen, D dimer, platelet, APTT and TT. PT, prothrombin time; PT-t, transformation of prothrombin time (the inverse of the difference between PT and the minimal PT of normal plasma); PCSK9, proprotein convertase subtilisin/Kexin type 9; CAD, coronary artery disease; BMI, body mass index; TG, triglyceride; TC, total cholesterol; HDL-C, high-density lipoprotein cholesterol; LDL-C, low-density lipoprotein cholesterol; HbA1C, hemoglobin A1C; hsCRP, high-sensitivity C-reactive protein; APTT, activated partial thromboplastin time; TT, thrombin time.

**Table S3. Cox regression analysis of PCSK9, PT-t status with MACEs.**

|  | **HR(95%CI)** | | | |
| --- | --- | --- | --- | --- |
| **Variables** | **Crude model** | ***p*** | **Adjusted model** | ***p*** |
| **PCSK9** |  |  |  |  |
| Low PCSK9 | 1 (Reference) | / | 1 (Reference) | / |
| High PCSK9 | 1.526(1.136,2.049) | 0.005 | 1.393(1.023,1.896) | 0.035 |
| **PT-t** |  |  |  |  |
| Low PT-t | 1 (Reference) | / | 1 (Reference) | / |
| High PT-t | 1.512(1.129,2.024) | 0.006 | 1.396(1.029,1.895) | 0.032 |
| **PCSK9+PT-t** |  |  |  |  |
| Low PCSK9+Low PT-t | 1 (Reference) | / | 1 (Reference) | / |
| Low PCSK9+ High PT-t | 1.415(0.891,2.246) | 0.141 | 1.369(0.854,2.193) | 0.192 |
| High PCSK9+ Low PT-t | 1.43(0.914,2.236) | 0.117 | 1.363(0.864,2.149) | 0.183 |
| High PCSK9+High PT-t | 2.049(1.381,3.041) | < 0.001 | 1.815(1.193,2.762) | 0.005 |

Adjusted model was adjusted for age, gender, CAD, diabetes, family history of CAD, TC, LDL, HbA1C, fibrinogen. MACEs, major adverse cardiovascular events; PCSK9, proprotein convertase subtilisin/kexin type 9; PT-t, transformation of prothrombin time (the inverse of the difference between PT and the minimal PT of normal plasma); prothrombin time; HR, hazard ratio; CI, confidence interval; CAD, coronary artery disease; TC, total cholesterol; HDL-C, LDL-C, low-density lipoprotein cholesterol; HbA1C, hemoglobin A1C. *p* < 0.05 suggested significant difference.

2583 patients with angina-like chest pain from October 2012 to April 2018 in Fu Wai hospital

2433 patients without lowering-lipid therapy were enrolled

patients with acute or heart failure, acute coronary syndrome, hematologic disorders, inflammatory disease, thyroid dysfunction, severe liver and/or renal insufficiency and malignant disease were excluded

2348 patients were followed-up for MACEs

patients completed the determination of PCSK9 and routine coagulation tests were excluded

91 patients lost follow-up

2293 patients were in final analysis

186 patients with MACEs

2107 patients without MACEs

**Figure S1. The flowchart of this study.** PCSK9, proprotein convertase subtilisin/kexin type 9; MACEs, major adverse cardiovascular events.
